# Additional lateral meniscus centralization reduces residual anterolateral rotatory instability after anterior cruciate ligament reconstruction and lateral meniscus repair

**DOI:** 10.1002/ksa.70200

**Published:** 2025-12-01

**Authors:** Ryota Seki, Tomomasa Nakamura, Nobutake Ozeki, Yusuke Nakagawa, Mai Katakura, Takashi Hoshino, Masaki Amemiya, Aritoshi Yoshihara, Hideyuki Koga

**Affiliations:** ^1^ Department of Joint Surgery and Sports Medicine Institute of Science Tokyo Tokyo Japan; ^2^ Center for Stem Cell and Regenerative Medicine Institute of Science Tokyo Tokyo Japan; ^3^ Department of Cartilage Regeneration Institute of Science Tokyo Tokyo Japan; ^4^ Clinical Center for Sports Medicine Institute of Science Tokyo Tokyo Japan

**Keywords:** anterior cruciate ligament reconstruction, anterolateral rotatory instability, centralization, lateral meniscus injury, triaxial accelerometer

## Abstract

**Purpose:**

This study aimed to examine the effects of lateral meniscus (LM) centralization on anterolateral rotational stability in patients with anterior cruciate ligament (ACL) and LM injuries. The hypothesis was that performing LM repair and centralization on knees with LM lesions would control anterolateral rotatory instability (ALRI).

**Methods:**

Twenty patients with ACL and LM injuries were enroled. Tibial acceleration in the pivot shift was quantified using a triaxial accelerometer at each preoperative and intraoperative time point (after ACL reconstruction (ACLR), LM repair and centralization) of double‐bundle ACLR. Pivot shift was assessed with the ACL graft tension provisionally fixed under two conditions that both the anteromedial bundle (AMB) and posterolateral bundle (PLB) grafts were temporarily fixed at 5 N each (A5P5) and at 10 N each (A10P10). The Kruskal–Wallis test was used to compare the tibial accelerations between each time points, and the Steel–Dwass test with Bonferroni correction was used for multiple comparisons. Also, patient factors associated with the results of a higher‐grade pivot shift compared to the healthy side after ACLR and LM repair were determined in a two‐group comparison.

**Results:**

Pivot shift acceleration was significantly decreased after ACLR (PreOP [8.3 ± 2.1 m/s^2^] vs. ACLR [5.1 ± 2.1 m/s^2^] in A5P5, *p* < 0.01 and [4.6 ± 1.5 m/s^2^] in A10P10, *p* < 0.01). However, the subsequent LM repair did not significantly reduce the pivot shift (ACLR [5.1 ± 2.1 m/s^2^] vs. LM repair [4.0 ± 0.9 m/s^2^] in A5P5, *p* = 0.09 and ACLR [4.5 ± 1.5 m/s^2^] vs. LM repair [3.5 ± 1.0 m/s^2^] in A10P10, *p* = 0.10). Additional centralization significantly controlled the residual pivot shift in most cases (ACLR [5.1 ± 2.1 m/s^2^] vs. Centralization [2.9 ± 1.0 m/s^2^] in A5P5, *p* < 0.01 and ACLR [4.5 ± 1.5 m/s^2^] vs. Centralization [2.8 ± 0.8 m/s^2^] in A10P10, *p* < 0.01).

**Conclusion:**

For patients who did not achieve sufficient rotational control after ACLR and LM repair, adding LM centralization further controlled ALRI in a relatively minimally invasive procedure.

**Level of Evidence:**

Level III.

**Abbreviations**
ACL: anterior cruciate ligament
ACLR: anterior cruciate ligament reconstruction
ALL: anterolateral ligament
ALRI: anterolateral rotatory instability
AMB: anteromedial bundle
CI: confidence interval
LET: lateral extra‐articular tenodesis
LM: lateral meniscus
LMPRT: lateral meniscus posterior root tear
MM: medial meniscus
OA: osteoarthritis
PLM: posterolateral bundle
PTS: posterior tibial slope
SSD: side‐to‐side difference

## INTRODUCTION

Anterior cruciate ligament (ACL) injuries often cause ALRI, which can remain after anterior cruciate ligament reconstruction (ACLR) [6, 16], affecting return to sports in athletes, performance levels and patient satisfaction, and possibly leading to osteoarthritis in the knee [3, 17]. Therefore, avoiding residual ALRI during ACL treatment is crucial.

Residual ALRI can be caused by ACL injury, younger age, preoperative knee hyperextension, preoperative high pivot shift grade, generalised joint laxity, and certain bone features around the knee, such as the distal femur and posterior tibial slope (PTS) [7, 13, 21, 36]. Studies report a correlation between LM injuries and ALRI [9, 10, 12, 14, 26]. Musahl et al. [26] reported an increased pivot shift phenomenon after LM resection in ACL‐deficient knees. Furthermore, lateral meniscus posterior root tears (LMPRT) exacerbate rotational instability [10], and LMPRT repair provides rotational stability [9]. There have also been reports of residual ALRI after ACLR following LM resection [12], and the pivot shift is controlled by LM repair [14]. In another cadaveric study, LM centralization to the knee with LM resection was confirmed to control rotational instability [29]. However, to date, no study has clinically examined the effect of LM centralization on controlling ALRI.

This study aimed to measure and evaluate anterolateral rotational stability during a pivot shift of the knee by using a triaxial accelerometer at each time point during surgery (preoperatively, after ACLR, LM repair, and centralization) in patients with ACL injury and LM tears. Based on previous findings, we hypothesised that adding LM centralization after ACLR and LM repair would further control ALRI.

## MATERIALS AND METHODS

### Patients

The crossover study was conducted. This study was approved by the Institutional Review Board in the Institute of Science Tokyo (protocol number: M2000‐1566), and we performed a crossover analysis. All the patients provided written informed consent to participate in the study. Patients with primary ACL injuries scheduled to undergo ACLR between February 2022 and October 2023 at the Institute of Science Tokyo Hospital were included.

LM injury was confirmed using arthroscopy during the operation. Patients with osteoarthritis greater than Kellgren–Lawrence grade 2, concomitant ligament injuries, open physis, or a history of injuries to the contralateral knees were excluded. Also, elite athletes with big pivot shifts or hyperextended knees were excluded from this study.

### Surgical procedure

#### Technique overview

Double‐bundle ACLR was performed in all patients by three attending surgeons. Arthroscopic examination was conducted through the standard anteromedial and anterolateral portals to confirm ACL and LM injuries, with subsequent treatment tailored to the extent of the injury. Medial meniscus (MM) injury, if present, was also treated according to the state of the injury.

#### Graft preparation and fixation

The semitendinosus tendon was harvested using an open‐loop stripper, divided into halves, and folded to yield two bundles: the AMB and the PLB. Femoral tunnel creation was performed without removing the remnant tissue using a behind‐remnant approach for double‐bundle ACLR [25]. Both the femoral and tibial tunnels were created anatomically. The outside‐in approach was used for femoral tunnel creation, whereas the tibial tunnel was created using the anteromedial surface. Grafts were inserted and securely fixed with EndoButton CL‐BTB (Smith & Nephew Endoscopy, Andover, MA) at the femoral end and the TensionLoc implant system (Arthrex, Naples, FL) at the tibial end, at 20° of flexion, ensuring equal tension between bundles.

#### LM repair

LM repair techniques vary based on the tear pattern and location. Tears confined to the posterior segment were sutured using the all‐inside technique, whereas those extending to the middle segment were sutured using an inside‐out technique with the Henning system (Stryker Japan, Tokyo, Japan). Vertical mattress sutures mostly repair longitudinal tears at the tear site on the tibial and/or femoral sides. Radial tears were repaired using tie‐grip sutures [30]. A pull‐out repair method was employed for LMPRT, involving bone tunnel creation at the attachment site from the anteromedial aspect of the proximal tibia, followed by a vertical mattress suture using a 2‐0 Fibre Wire (Arthrex, Naples, FL) and a racking hitch knot with two suture tapes to hold the torn edge of the LM posterior root. Sutures were passed through the tunnel and fixed using the TensionLoc implant system.

#### Centralization technique

Centralization was performed in cases of LM injury according to the original method [18]. An arthroscopic view from the anterolateral portal was used to create a midlateral portal just proximal to the LM and 5 mm anterior to the popliteal hiatus, allowing for the insertion of the suture anchors. A 1.8‐mm Q‐FIX all‐suture anchor with a two‐strand #2 MAGNUMWIRE suture (Smith & Nephew Endoscopy, Andover, MA) was inserted at the lateral edge of the lateral tibial plateau just anterior to the popliteal hiatus. The extruded LM was easily displaced and protected by using a cannula for anchor insertion. A Micro SutureLasso with a small curve and nitinol wire loop (Arthrex, Naples, FL) was introduced through the midlateral portal. The tip of the Micro SutureLasso penetrated the capsule from superior to inferior along the margin of the meniscus. The wire loop was advanced into the knee joint, and the Micro SutureLasso was retrieved. A suture grasper was inserted through the anteromedial portal, and the wire loop and one strand of the MAGNUMWIRE were retrieved. The retrieved MAGNUMWIRE strand was threaded into the wire loop, and the opposite limb of the wire loop was pulled to pass the MAGNUMWIRE from inferior to superior. This process was repeated using another strand of MAGNUMWIRE to create the mattress suture configuration. A second Q‐FIX all‐suture anchor was placed 1 cm anterior to the first anchor at the lateral edge of the lateral tibial plateau, and the same steps were repeated. Finally, the sutures were tied through the midlateral portal using a self‐locking sliding knot, and the displaced meniscus was centralised.

When the lateral joint capsule was exposed using the conventional inside‐out meniscus suture repair technique, capsulodesis was performed to centralise the extruded meniscus [32]. Capsulodesis was performed directly using 1.8‐mm Knotless FiberTak Soft Anchors (Arthrex). A spinal needle (23 G) was inserted beneath the meniscus from outside the joint to ensure appropriate anchor placement. The anchor was placed at the edge of the tibial plateau through the skin incision used for the meniscus suture. Three anchors were inserted, spaced 1 cm apart. The repair suture from the second anchor was threaded into the shuttling suture loop of the first anchor, and the shuttling suture of the first anchor was gently pulled to pass the repair suture through the first anchor, which was then tightened. Similarly, the repair suture from the third anchor was passed through the shuttling suture loop of the second anchor, and the shuttling suture of the second anchor was pulled to pass the repair suture through the second anchor, which was also tightened. At this point, the capsule was significantly stabilised, allowing the repair suture of the first anchor to be removed.

### Measurement of tibial acceleration

To objectively quantify the pivot shift test, we measured tibial acceleration during the pivot shift using a kinematic rapid assessment (KiRA) triaxial accelerometer (Orthokey, Lewes, DE; Figure 1a). This system establishes a wireless connection with a tablet via Bluetooth to conduct knee movement analysis. Before testing, the device was attached to the anterolateral aspect of the proximal tibia and positioned between the tibial tuberosity and Gerdy's tubercle with a belt around it. The device measured the acceleration of the tibia during the pivot shift tests (m/s^2^) and calculated the difference between the maximum (a_max_) and minimum (a_min_) values (a_range_: a_max_–a_min_), indicating the degree of subluxation during the pivot shift phenomenon. Five consecutive pivot shift tests were performed, and the average of the three middle values was calculated. Two attending surgeons performed all the tests. By this method, tibial acceleration was measured on the healthy (CTRL) and affected sides under anaesthesia preoperatively and intraoperatively after ACLR (ACLR alone group), LM repair (LM repair group) and additional centralization (LM repair + centralization group), if repair‐required LM injuries were observed during arthroscopic examination. Intraoperative measurements were conducted using the Graft Tensioning System (ConMed Linvatec, Largo, FL) with both the AMB and PLB grafts temporarily fixed at 5 N each (A5P5) at 20° knee flexion and at 10 N (A10P10; Figure 1b). The acceleration at each time point was calculated and compared with CTRL.

**Figure 1:**
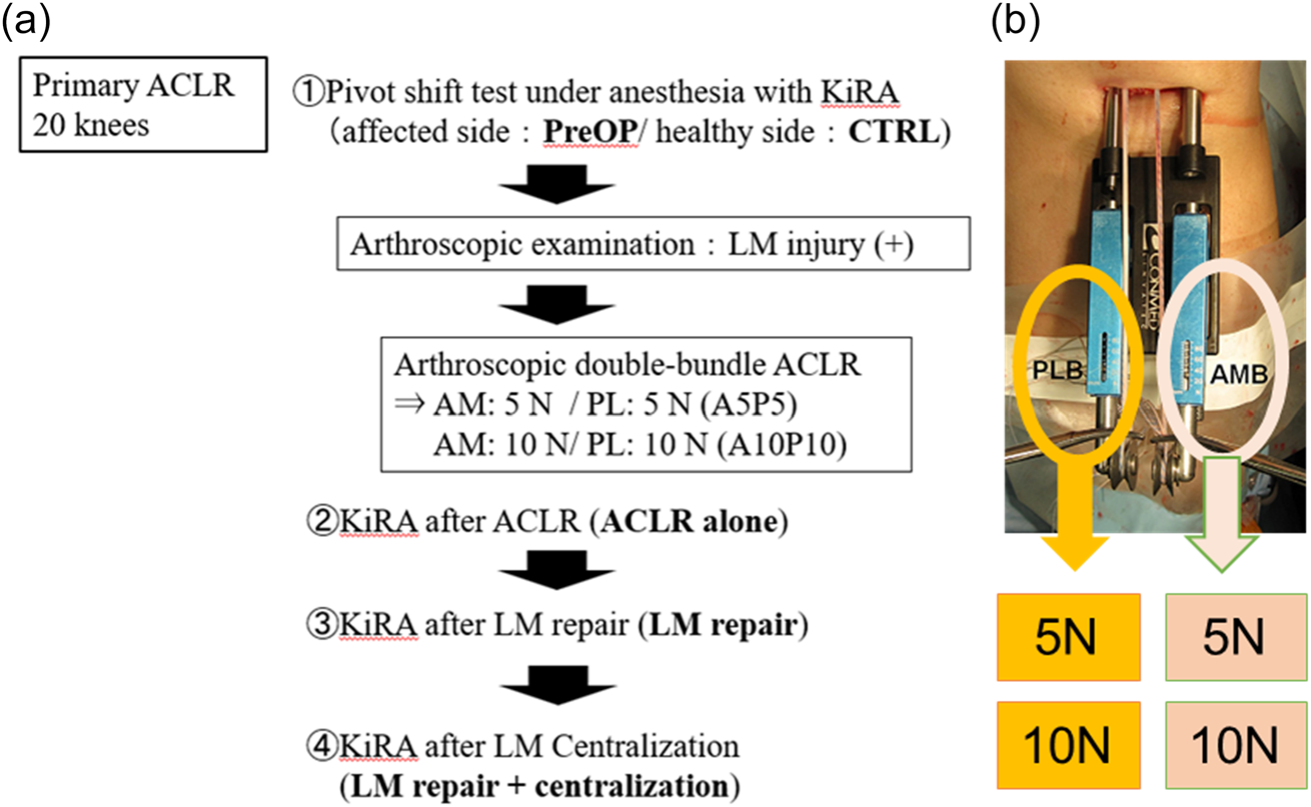
Study protocol. (a) Pivot shift tests on the affected side (PreOP) and healthy side (CTRL) under anaesthesia were performed using kinematic rapid assessment (KiRA) to measure the acceleration of the tibia. In the case of ACL with LM injury, acceleration was measured at each time point (after ACLR, after LM repair, and after LM centralization). (b) Pivot shift measurements were performed under two conditions using a graft‐tensioning system. AMB and PLB were fixed at 5 N each (A5P5) and 10 N each (A10P10), respectively. ACLR, anterior cruciate ligament reconstruction; AMB, anteromedial bundle; LM, lateral meniscus; PLB, posterolateral bundle.

### Data collection

To assess residual ALRI factors, patient background data, such as age, sex, preoperative period, preoperative pivot shift grade, KT‐1000 arthrometer results, presence of MM injury, type of LM injury, the amount of preoperative LM extrusion and posterior tibial slope (PTS) were collected. The pivot shift test was performed under anaesthesia by an attending surgeon, and tibial acceleration was recorded with a KiRA triaxial accelerometer. Anterior knee laxity was measured preoperatively by an attending surgeon using a KT‐1000 arthrometer under anaesthesia. This study evaluated the values of the healthy side and side‐to‐side differences in the manual maximum pull. Generalised joint laxity was scored by examining eight joints (shoulder, elbow, hand, thumb, spine, hip, knee, and ankle) based on previous studies [34]. If laxity was observed in either the left or right joint, the joint scored one point, for a total score of eight points. The amount of LM extrusion was measured using preoperative MRI. The length of the extruded meniscus was measured from a line connecting the lateral edges of the femoral and tibial articular surfaces at the slice of just anterior to the popliteal hiatus on the coronal plane. PTS was measured using sagittal magnetic resonance imaging (MRI) at the medial and lateral angles [42].

### Statistical analysis

Statistical analysis was performed using SPSS software (version 29.0; SPSS, Chicago, IL). After assessment of the normal distribution of the data using the Shapiro–Wilk test, the Kruskal–Wallis test was used to compare the tibial accelerations between all groups, and the Steel–Dwass test with Bonferroni correction was used for multiple comparisons. To evaluate the risk factors for residual ALRI after LM repair, the Mann–Whitney *U* test or Fisher's exact test was performed, due to the lack of normal distribution of the data (assessed by the Shapiro–Wilk test), to compare each factor between the residual and controlled ALRI groups. Cases in which the pivot shift was controlled with ACLR and LM repair were compared with those in which the pivot shift was controlled with the addition of centralization using a chi‐square test. Statistical significance was set at *p* < 0.05 for all analyses. Preoperative period was expressed as median, and the other values of data were expressed as mean ± standard deviation (SD). An a priori power analysis was performed using G‐power 3.1 software (Kiel University, Kiel, Germany). Based on a previous study [14], the observed effect size for the alteration in pivot shift acceleration before and after LM repair was approximately 0.80. Consequently, a minimum sample size of 19 patients was determined to be necessary to achieve a statistical power of 0.80, maintaining an alpha level of 0.05.

## RESULTS

### Patient characteristics

Twenty patients were included in the study based on the inclusion criteria (Table A1). The patient ages ranged from 14 to 54 years, with a mean age of 29 years; there were nine females and 11 males. The preoperative period ranged from 1 to 480 months, with a median of 2.8 months. The general joint laxity scores ranged from 0 to 6, with a mean score of 1.8. According to the KT‐1000 arthrometer measurements, the mean values of the healthy side were 8.5 mm, ranging from 4 to 14 mm, and the mean values of the side‐to‐side difference were 5.6 mm, ranging from 3 to 11 mm. In addition, 13 patients had MM injuries, all of whom underwent repair. The observed types of LM injuries were longitudinal tears in 12 cases; horizontal, radial, and flap tears in one case each; and posterior root tears in five cases.

### Tibial acceleration

Tibial acceleration was significantly decreased by ACLR in both A5P5 (5.1 ± 2.1 m/s^2^, 95% confidence interval [CI]:4.1–6.1), and A10P10 (4.5 ± 1.5 m/s^2^, 95% CI: 3.8–5.2; Figure 2 and Table 1). However, in the A5P5 condition, the mean acceleration after ACLR tended to be higher than that of CTRL (4.7 ± 1.0 m/s^2^, 95% CI: 4.2–5.2). In addition, tibial acceleration decreased after LM repair, but not significantly (4.0 ± 0.9 m/s^2^, 95% CI: 3.6–4.4 in A5P5, *p* = 0.09, 3.5 ± 1.0 m/s^2^, 95% CI: 3.0–4.0 in A10P10, *p* = 0.10). Conversely, additional centralisation (2.9 ± 1.0 m/s^2^, 95% CI: 2.4–3.4 in A5P5, 2.8 ± 0.8 m/s^2^, 95% CI: 2.4–3.2 in A10P10) resulted in a further significant decrease in acceleration (*p* < 0.05 in A5P5 and A10P10).

**Figure 2:**
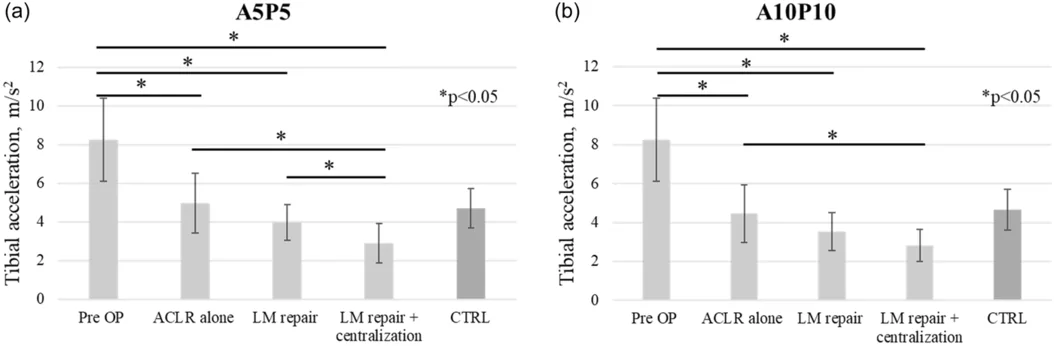
Tibial acceleration at each time point (A5/P5, A10/P10). Tibial acceleration at each timepoint in (a) A5P5 and (b) A10P10. ACLR reduced tibial acceleration compared with that of the preoperative knee. Although LM repair alone after ACLR did not significantly reduce tibial acceleration, the addition of centralization significantly reduced acceleration. ACLR, anterior cruciate ligament reconstruction; LM, lateral meniscus.

**Table 1:** Tibial acceleration (m/s^2^) at each time point for each case.

(A) A5P5					
Case no.	PreOP	ACLR alone	LM repair	LM repair + centralization	CTRL
1	**9.5**	**5.2**	3.7	1.9	4.3
2	**8.6**	5.2	3.2	2.5	5.2
3	**6.2**	5.4	4.2	2.6	5.7
4	**9.2**	4.2	1.9	1.1	4.5
5	**8.4**	**5.8**	5.0	3.2	5.5
6	**7.1**	**5.6**	**4.8**	**3.6**	2.8
7	**9.9**	4.7	4.0	3.0	5.4
8	**10.1**	3.0	3.2	2.7	4.8
9	**7.4**	4.7	4.9	2.1	5.4
10	**7.7**	**5.3**	2.8	2.5	4.4
11	**5.3**	**5.4**	**4.8**	3.3	3.6
12	**6.4**	4.8	4.0	3.4	5.8
13	**8.0**	3.7	**4.8**	3.0	4.0
14	**11.1**	4.1	3.6	2.6	4.4
15	**13.4**	**10.4**	5.0	4.8	6.8
16	**3.2**	2.5	**2.8**	2.3	2.5
17	**9.9**	4.3	3.0	2.1	4.8
18	**7.7**	**4.4**	**4.7**	2.8	4.1
19	**7.5**	**6.0**	5.1	4.7	5.4
20	**8.7**	**7.5**	4.7	4.2	5.2

(B) A10P10					
Case no.	PreOP	ACLR alone	LM repair	LM repair + centralisation	CTRL
1	**9.5**	**4.8**	2.2	2.5	4.3
2	**8.6**	4.5	3.5	2.4	5.2
3	**6.2**	4.0	4.0	2.7	5.7
4	**9.2**	3.7	2.6	1.4	4.5
5	**8.4**	5.1	4.2	2.6	5.5
6	**7.1**	**3.7**	**4.3**	**3.4**	2.8
7	**9.9**	3.8	3.4	2.8	5.4
8	**10.1**	2.7	2.7	2.8	4.8
9	**7.4**	**5.6**	4.0	2.6	5.4
10	**7.7**	4.4	2.7	2.4	4.4
11	**5.3**	**4.1**	3.3	3.6	3.6
12	**6.4**	4.4	4.3	3.2	5.8
13	**8.0**	3.8	3.4	3.6	4.0
14	**11.1**	4.2	3.4	2.5	4.4
15	**13.4**	**9.7**	6.3	5.4	6.8
16	**3.2**	2.3	2.3	1.8	2.5
17	**9.9**	4.6	2.7	2.3	4.8
18	**7.7**	**4.8**	**4.4**	3.0	4.1
19	**7.5**	**5.8**	**5.6**	3.9	5.4
20	**8.7**	**6.4**	4.2	4.4	5.2

*Note*: Values in bold represent tibial accelerations greater than the healthy knee (CTRL). (A) In the A5P5 and (B) A10P10 conditions, five and three cases, respectively, showed residual ALRI after LM repair. Additional centralization further controlled tibial acceleration under both conditions.

Abbreviations: ALRI, anterolateral rotatory instability; LM, lateral meniscus.

### Residual ALRI after ACLR and LM repair

To indicate cases of residual ALRI even after LM repair, the tibial acceleration at each timepoint was shown in Table 2. Five cases of A5P5 and three cases of A10P10 were observed, in which tibial acceleration was not well controlled compared to that in the CTRL group after LM repair. In each condition, cases with residual ALRI after LM repair (Residual ALRI) and cases in which ALRI was controlled (Controlled ALRI) were compared (Table 2). The results showed that all patients with residual ALRI were male (*p* = 0.04 in the A5P5 condition), and in the A5P5 condition, the preoperative tibial acceleration was significantly lower in the residual ALRI group (6.3 ± 2.0 m/s^2^, 95% CI: 3.8–8.7 in controlled ALRI group, 8.9 ± 1.9 m/s^2^, 95% CI: 7.9–10.0 in residual ALRI group, *p* = 0.03).

**Table 2:** Patient characteristics of residual ALRI after ACLR and LM repair in each provisional condition.

A5P5			
	Residual ALRI (*n* = 5)	Controlled ALRI (*n* = 15)	*p* value
Gender female/male	0/5	9/6	**0.04**
Age, years	24 (17–38)	31 (14–54)	0.40
Preoperative period, months	2.5 (1–4)	3 (2–480)	0.30
MM injury, yes/no	2/3	11/4	0.29
General joint laxity	1.6 (0–5)	1.7 (0–6)	1.00
Preoperative KT‐1000 SSD, mm	5.3 (3.5–7.0)	5.7 (3.0–11.0)	0.87
Preoperative tibial acceleration, m/s^2^	3.4 (2.5–4.1)	5.2 (4.3–6.8)	**0.03**
Medial posterior tibial slope, °	6.4 (4.2–10.5)	4.9 (1.6–11.6)	0.10
Lateral posterior tibial slope, °	7.3 (5.3–10.8)	7.0 (4.8–11.5)	0.74

A10P10			
	Residual ALRI (*n* = 3)	Controlled ALRI (*n *= 17)	*p* value
Gender female/male	0/3	9/8	0.22
Age, years	24 (17–30)	29.9 (14–54)	0.69
Preoperative period, months	3 (3–4)	2.5 (2–480)	0.92
MM injury, yes/no	1/2	12/5	0.27
General joint laxity	3.3 (2–5)	1.4 (0–6)	0.09
Preoperative KT‐1000 SSD, mm	5.7 (5.0–7.0)	5.6 (3.0–11.0)	0.77
Preoperative tibial acceleration, m/s^2^	4.1 (2.8–5.4)	4.8 (2.5–6.8)	0.26
Medial posterior tibial slope, °	5.4 (4.2–6.7)	4.1 (1.6–11.6)	0.48
Lateral posterior tibial slope, °	7.2 (6.8–8.0)	7.0 (4.8–11.5)	0.55

*Note*: The value of the preoperative period was expressed as median (range), and the other values were expressed as mean (range). *p* Value from Mann–Whitney *U* test or Fisher's exact test. Values in bold indicate statistical significance.

Abbreviations: ACLR, anterior cruciate ligament reconstruction; ALRI, anterolateral rotatory instability; LM, lateral meniscus; SSD, side‐to‐side difference.

Four of the five cases of residual pivot shift after ACLR and LM repair in A5P5 were controlled by additional centralization. In A10P10, all three cases of residual pivot shifts were controlled after centralization (Table 1).

## DISCUSSION

The most important finding of this study is that LM centralization contributed to controlling residual ALRI after ACLR and LM repair. ALRI persists due to LM injury, and LM repair controls ALRI. Forkel et al. [9] reported that repairing the LMPRT controlled the pivot shift. LMPRT has also been reported to contribute to ALRI after lateral meniscectomy. In this study, LM repair did not adequately control the pivot shift in some cases. However, the LM centralization was found to further control the pivot shift.

The original purpose of the centralization technique was to repair or prevent meniscal extrusion and prevent the progression of osteoarthritis (OA) [18, 20], and favourable 2‐year postoperative clinical outcomes have been reported [19]. Koga et al. reported that the lateral joint space widened on 2‐year postoperative radiographs after LM centralization. In the present study, anterolateral rotational stability was obtained secondary to the procedural effect of capsule tightening by centralization or capsulodesis.

In addition, this study was performed with the tension of temporary fixation of the ACL grafts under two conditions, both at a tension lower than the actual fixation tension. In a study conducted prior to this one, (unpublished data), the minimum tension of the ACL graft required to control the pivot shift was found, which was reduced to 10 N by LM repair. Therefore, in this study, the tension of each graft was set below 10 N to minimise the influence of the ACL on anterolateral stability. Another point of view, the ACL typically loosens appropriately over the postoperative course, and in this study, the provisional fixation tension was set to simulate that state. In three of the 20 patients, the pivot shift was larger than that of the healthy side, even under 10 N of the graft tension after LM repair, but this was controlled in all cases by the addition of centralization.

ACLR with anterolateral ligament (ALL) reconstruction or lateral extra‐articular tenodesis (LET) has been reported as a method for controlling ALRI [2, 5, 11, 22, 39, 41]. Although these methods have been reported to provide good stability for patients with residual ALRI after ACLR [2, 39, 41], they are relatively invasive, requiring the sacrifice of healthy tendons and the creation of additional skin incisions or bone tunnels. Postoperative problems have also been reported [5, 11], such as residual lateral pain and a prolonged return to physical activity. Another concern is that, depending on the tension of the fixation, the load on the lateral compartment of the knee may increase excessively, increasing the possibility of OA progression. In contrast, the results of this study suggest that LM repair and centralization in ACL‐injured patients with LM injuries may achieve anterolateral stability while maintaining meniscal function and possibly preventing future OA progression with minimally invasive procedures.

In this study, all patients who underwent residual ALRI after ACLR and LM repair were male. Batty et al. [4] reported that the male sex was a risk factor for residual postoperative pivot shift in 618 patients with ACL injuries, which is consistent with the results of our study. Conversely, some studies have reported a higher‐grade pivot shift in females [24, 33]; the MOON cohort reported by Magnussen et al. [24] was one of the largest studies, showing a significantly higher‐grade pivot shift in females on preoperative examination after ACL injury. In other studies, no significant differences between sexes have been reported [7, 23, 31, 36]. It is difficult to explain this sex difference because of the small number of patients included in this study.

Risk factors for residual ALRI include preoperative hyperextended knees, high pivot shift grade, general joint laxity, and characteristic bone morphology such as steep PTS [4, 13, 15, 21, 24]. In the present study, in the A5P5 condition, in which the influence of the reconstructed ACL was minimised, the preoperative pivot shift was significantly larger in patients with residual ALRI after LM repair alone. As mentioned previously, the number of cases was small; therefore, a two‐group comparison was performed. It is possible that a significant difference between the groups could be obtained for other factors, as well as an increase in the number of cases.

The MM plays an essential role in anterior knee stability [1, 35] in ACL‐deficient knees, but its contribution to rotational stability is controversial [24, 26, 27]. Some studies reported that ramp lesions contribute to rotational stability [8, 37]. DePhillipo et al. [8] and Stephen et al. [37] reported that rotational stability was not restored after ACLR alone, but was restored after ACLR combined with ramp repair. On the other hand, Tokura et al. [38] reported that ramp lesions were not associated with increased tibial acceleration during the pivot shift test. In the present study, six patients had ramp lesions that were all repaired, and the pivot shift was controlled in all but one case after ACLR.

This study has several limitations. First, the pivot shift test was performed under anaesthesia, and it was difficult to evaluate the effect of ALRI control in the mid‐to‐long‐term postoperative periods in the same manner. In the future, it will be necessary to compare the clinical outcomes, including the level of return to sports and patient satisfaction. Second, this study did not include high‐risk patients with residual ALRI. General indications for LET or ALL reconstruction are based on previous reports [13, 40]. They were defined as ACL reinjury cases, competitive athletes with a high‐grade pivot shift, and hyperextended knees of 5° or more under anaesthesia. Therefore, the effect of centralization on the control of ALRI in high‐risk cases is unclear. However, it is important to note that there have been cases in which ALRI could not be well controlled with ACL and LM repair alone. And the residual ALRI can be controlled through additional centralization. In future studies, comparisons with high‐risk patients are required. Third, two attending surgeons performed the pivot shift test manually, and the study itself was not blinded. However, both surgeons were experienced and well trained, and the procedure was performed according to the standardised methods of previous studies [14, 15, 28].

## CONCLUSION

For patients in whom rotational instability persists after ACLR and LM repair, LM centralization offers further control over ALRI through a relatively minimally invasive procedure.

## AUTHOR CONTRIBUTIONS

Hideyuki Koga and Tomomasa Nakamura conceived and planned the experiments. Ryota Seki, Hideyuki Koga, Tomomasa Nakamura, Nobutake Ozeki, Yusuke Nakagawa, Mai Katakura, Takashi Hoshino, Masaki Amemiya and Aritoshi Yoshihara carried out the experiments. Ryota Seki, Hideyuki Koga, Tomomasa Nakamura, Nobutake Ozeki, Yusuke Nakagawa, Mai Katakura, Takashi Hoshino, Masaki Amemiya and Aritoshi Yoshihara contributed to the interpretation of the results. Ryota Seki took the lead in writing the manuscript. Tomomasa Nakamura finalised the manuscript. All authors provided critical feedback and helped shape the research, analysis and manuscript.

## CONFLICT OF INTEREST STATEMENT

The authors declare no conflicts of interest.

## ETHICS STATEMENT

This study was approved by the Institutional Review Board in the Institute of Science Tokyo (protocol number M2000‐1566). All the patients provided written informed consent to participate in the study.

## Supporting information

Supporting information.

Supporting information.

## Data Availability

Data openly available in a public repository that issues datasets with DOIs.
