# Supplementary material for: Additional lateral meniscus centralization reduces residual anterolateral rotatory instability after anterior cruciate ligament reconstruction and lateral meniscus repair
Source: Knee Surg Sports Traumatol Arthrosc. 2025 Dec 1;34(11):3980–8. doi: 10.1002/ksa.70200 (PMC13703934; doi:10.1002/ksa.70200)
Supplement: Supplementary file 1 [file KSA-34-3980-s001.docx]

**Appendix 1**

**Table A1. Patient demographic data (n=20)**

| Age, years | | 29±12 (14–54) |
| --- | --- | --- |
| Gender female/male, n | | 9/11 |
| Preoperative period, months | | 2.8 (1–480) |
| General joint laxity | | 1.8±1.9 (0–6) |
| KT-1000 (healthy side), mm | | 8.5±2.6 (4–14) |
| KT-1000 (SSD), mm | | 5.6±2.0 (3–11) |
| MM injury, n | | 13 (Repair 13) |
| LM injury type, n | Longitudinal | 12 |
|  | Horizontal | 1 |
|  | Radial | 1 |
|  | Flap | 1 |
|  | Posterior root | 5 |
| LM extrusion length, mm | | 2.8±0.9 (1.0-5.0) |

The values of the preoperative period are expressed as the median (range) and the other values are expressed as the mean±SD (range). SSD, side-to-side difference; MM, medial meniscus; LM, lateral meniscus.
